# Functional evaluation and practice survey to guide purchasing of intravenous cannulae

**DOI:** 10.1186/1471-2253-13-49

**Published:** 2013-12-24

**Authors:** Stanley Tay, Brian Spain, Kirstie Morandell, Jesse Gilson, Laurence Weinberg, David Story

**Affiliations:** 1 Department of Anaesthesia, Royal Darwin Hospital, Tiwi, NT 0810, Australia; 2 Department of Anaesthesia, Austin Hospital, Heidelberg, VIC 3084, Australia; 3 Centre for Anaesthesia, Perioperative and Pain Medicine, The University of Melbourne, Parkville, VIC 3053, Australia

**Keywords:** Intravenous cannula, Purchasing guide

## Abstract

**Background:**

There are wide variations in the physical designs and attributes between different brands of intravenous cannulae that makes product selection and purchasing difficult. In a systematic assessment to guide purchasing, we assessed two cannulae – Cannula P and I. We proposed that the results of in-vitro performance testing of the cannulae would be associated with preference after clinical comparison.

**Methods:**

We designed an observer-blinded randomised head-to-head trial between the 18, 20 and 22 gauge versions of Cannula P and I. Our primary end-point was pressure (mmHg) generated during various flow rates and our secondary end-point was the force (Newton) required to slide the catheter away from the needle. This was followed by a prospective electronic survey following a two-week clinical trial period.

**Results:**

The mean difference in resistance between Cannula P and I was: 307 mmHg.L^-1^.hr^-1^ (95% CI: 289–325, p < 0.001) for 22G; 135 mmHg.L^-1^.hr^-1^ (95% CI: 125–144, p < 0.001) for 20G; and 27 mmHg.L^-1^.hr^-1^ (95% CI: 26–28, p < 0.001) for 18G. The mean difference in the force needed to displace the catheter away from its needle was: 1.41 N (95% CI: 1.09-1.73, p < 0.001) for 22G; 0.19 N (95% CI: -0.04-0.41, p = 0.12) for 20G; and 1.96 N (95% CI: 1.40-2.52, p < 0.001) for 18G. After a trial period, all 16 anaesthetist who had used both cannulae preferred Cannula I to P.

**Conclusions:**

The evaluation process described here could help hospitals improve efficient product selection and purchasing decisions for intravenous cannulae.

## Background

There are millions of peripheral intravenous cannulae (IVC) used each year around the world [1]. Purchasing decisions to deliver economic savings should be based on a cost-effectiveness analysis of the different types of IVC available. This analysis has to incorporate in order of importance, the safety in preventing needle-stick injuries, efficacy in achieving successful cannulation and the unit cost. Individual experience and preference will also play a role. This process can be difficult and underlines the importance of standardisation to facilitate familiarity and avoid unwanted duplication.

Currently designs vary greatly between the manufacturers. This is despite manufacturing standards set by the International Organization of Standardization (ISO). Currently the standards described in ISO 10555–1 and 5 on the physical design of the needle (needle tube) of an IVC do not have specific requirements other than to permit flashback [2,3]. An appropriate design is important as it affects the success rates of cannulation [4-6]. Furthermore regulatory approval from organisations such as the Therapeutic Goods Administration in Australia or the Medical Device Directive in the European Union are often based on medical equipment meeting ISO standards. Therefore as long as these limited standards are met, manufacturers are allowed to market these devices without being required to provide evidence on its efficacy [7].

We therefore proposed that the results of in-vitro performance testing, comparing the resistance in the needle as a surrogate measure of flashback, and the force needed to displace the catheter from its needle as a surrogate measure of static friction, would be associated with a favourable opinion from medical staff following a trial period.

## Methods

This project was conducted at the Royal Darwin Hospital, a University teaching hospital in Darwin, Australia. The decision to clinically trial both Cannula P (Polywin Safety®, Multigate Medical Devices Pty Ltd, Yennora, NSW, Australia) and Cannula I (Introcan Safety®, B. Braun Melsungen AG, Melsungen, Germany) over a two-week period had been approved by the Procurement Review Committee. Therefore, waiver of informed consent from patients was allowed by the Menzies School of Health Research Ethics Committee (QAAR-2013-2000) as our project only involved two laboratory tests and a prospective electronic survey of anaesthetists. All responses were anonymous. Both IVC employ the same passive safety mechanism (automatic deployment of safety needle guard following withdrawal of needle from hub) to avoid needle-stick injures.

**Figure 1:**
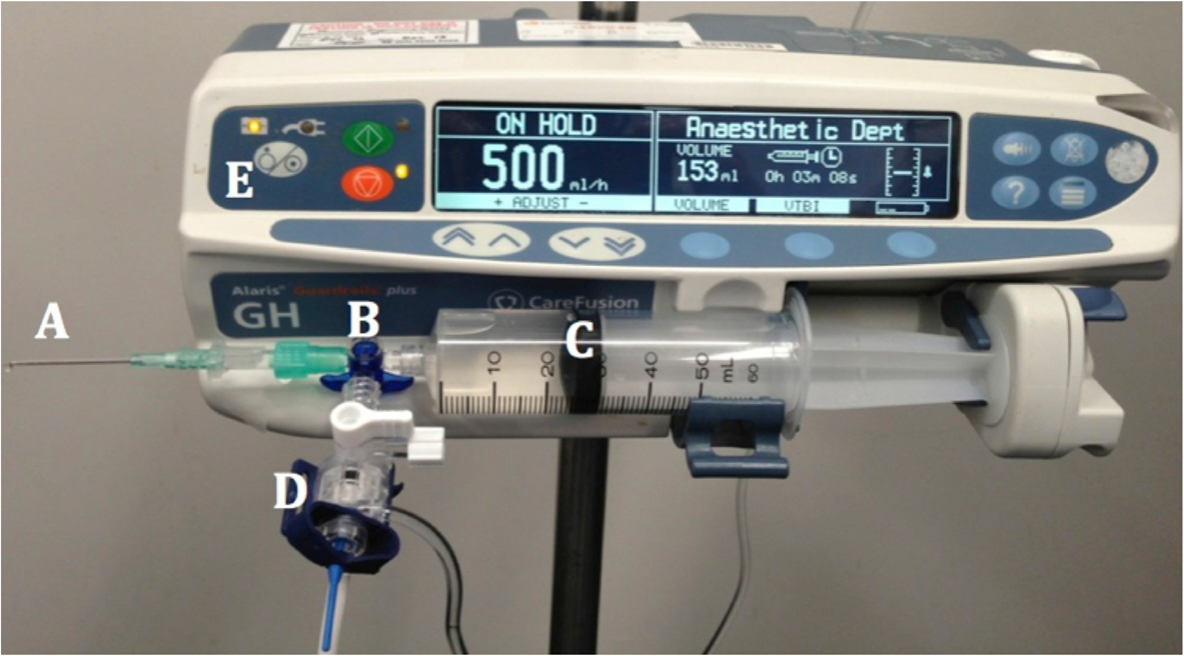
**Setup up to indirectly measure resistance by recording the pressure generated through an intravenous cannula’s needle at various flow rates.** Consisting of **A**: intravenous cannula; **B**: 3-way tap; **C**: 50 ml syringe; **D**: Pressure transducer; **E**: Syringe driver.

For our laboratory tests, we designed an observer-blinded randomised head-to-head trial between the 18, 20 and 22 gauge versions of Cannula P and Cannula I. Our primary end-point was pressure (mmHg) generated during various flow rates as an indirect measure of resistance in the needle tube. This is because the change in pressure measured is directly proportional to the resistance of the needle as described by Hagen-Poiseuille’s equation: ΔP = 8.l.η.Q ÷ π.r [4] (P = Pressure; l = length; η = viscosity; Q = Flow; r = radius). To measure this, we removed each IVC’s vent fitting and attached the needle directly onto a 50 ml luer lock syringe (Terumo Medical Corporation, Somerset, New Jersey, United States) filled with distilled water via a 3-way tap. We used distilled water instead of normal saline as this is the same fluid that is recommended by ISO to test flow rates through catheters. An arterial pressure transducer was then connected to the third port as shown in Figure 1. Pressures were displayed real-time on a Philips monitor. A syringe driver (Alaris, Carefusion, San Diego, California, United States) serviced and calibrated in February 2013, was used to deliver set flow rates in 100 ml.hr^-1^ increments up to 1000 ml.hr^-1^ or until the pressures measured exceeded the limit of 360 mmHg.

A preliminary estimate of sample size was based on a non-inferiority margin of 5% in the change in pressure for a given change in flow rate. This was conservatively set within the margin of errors described by ISO 10555–1 and 5 for prescribed parameters. Based on our pilot study of the 18G Cannula I, this was a change in the mean pressure of ±2.2 mmHg (SD = 2.1). With a type I error of 0.05 and a type II error of 0.1, we would be required to test 10 cannulae per group with two-sided significance (60 measurements in total). The order in which these were tested was randomised using a table of random numbers. The blinded observer then recorded the measurements. To ensure stability, each measurement was preceded by the zeroing of the transducer with the 3-way tap open to all three ports.

**Figure 2:**
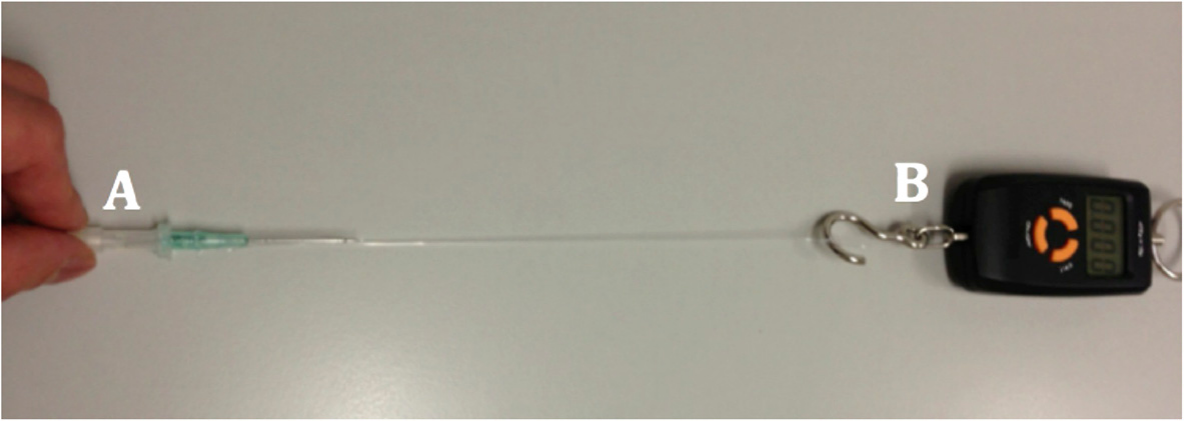
**Setup up to indirectly measure static friction by recording the force required to displace the catheter away from the needle.** Consisting of **A**: intravenous cannula; **B**: electronic spring scale.

Our secondary end-point was the force (Newton) required to slide the catheter away from the needle as an indirect measure of its static friction. To measure this, we attached each catheter to an electronic spring scale as shown in Figure 2. We again measured 10 IV cannulae of each gauges from each brand (60 measurements in total) and randomised the order using a table of random numbers. Each catheter was pulled at approximately 0.1 N.s^-1^ until the catheter started to slide away from the needle. The force at which this occurred was then recorded by the blinded observer.

### Statistical analyses

A computerized statistical package (Small Stata 12.0 for Mac, StataCorp, Texas, United States) was used for data analysis. Linear regression analysis was used to model the change in pressure for a given change in flow rate, with the intercept set at 0 for each IVC. The mean values in each group were compared by a two-tailed, unpaired heteroscedastic Student’s *t*-test. P values of < 0.05 were considered statistically significant. Variance was reported by using standard deviations (SD), relative standard deviations (RSD) and 95% confidence intervals (95% CI).

## Results

**Figure 3:**
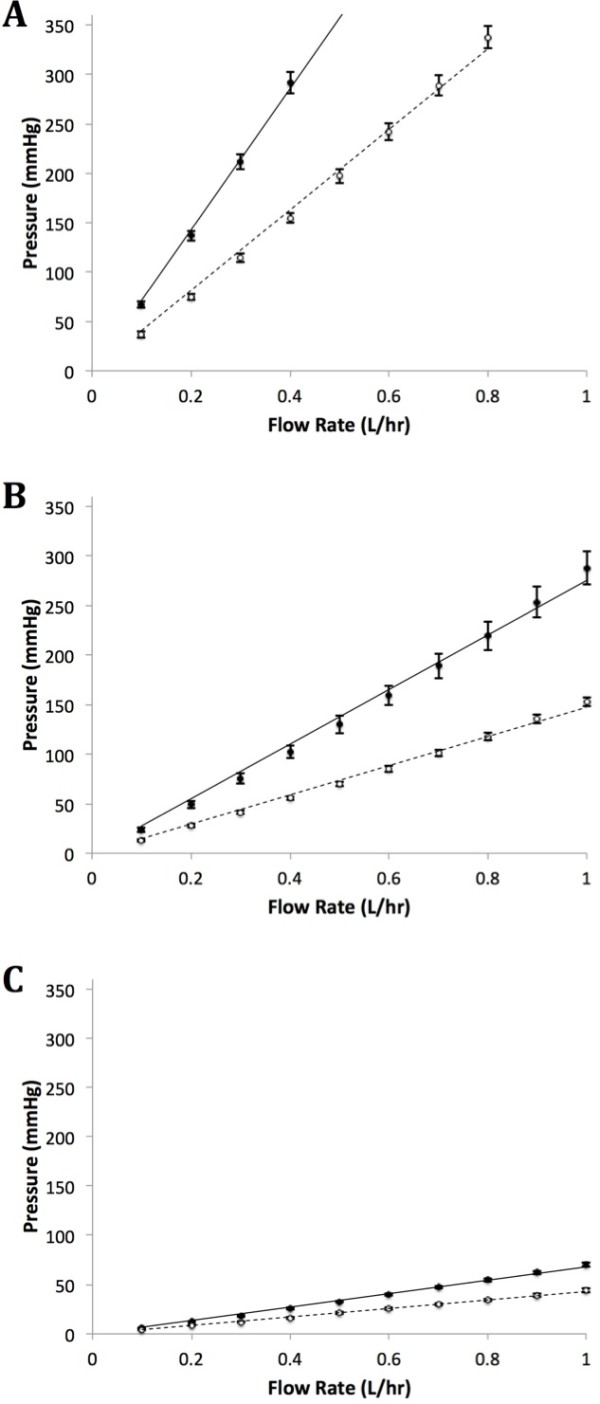
**Pressure (mmHg) measured for a given flow rate (L.hr**^**-1**^**) as an indirect measure of resistance in Cannula (•) P and (*ο*) I: 22G (A), 20G (B) and 18G (C).** Only the mean pressure from the 10 measurements at each designated flow rates are plotted. Standard deviations are represented by error bars. Trends lines are also drawn for Cannula () P and () I.

Cannula P consistently exhibited higher resistance in its needle compared to Cannula I. These results are summarised in Figure 3 and Table 1. The resistance in the 22G Cannula P was 73% higher than Cannula I (p < 0.001), 89% higher in the 20G (p < 0.001) and 61% higher in the 18G (p < 0.001). With respect to precision, all except the 20G Cannula had a relative standard deviation less than 5%. A pressure of 0 mmHg was recorded through the various flow rates when using the 3-way tap in isolation to confirm it did not have any significant effect on our measurements.

**Table 1:** Measurements of resistance through the needle in Cannula P and I

	**Cannula P**	**Cannula I**	**Difference**	**p-value**
22G resistance; mmHg.L^-1^.hr^-1^	728 (3.7)	421 (3.6)	307 [289 to 325]	2 × 10^-14^
20G resistance; mmHg.L^-1^.hr^-1^	287 (6.3)	152 (3.1)	135 [125 to 144]	3 × 10^-10^
18G resistance; mmHg.L^-1^.hr^-1^	71 (2.4)	44 (4.7)	27 [26 to 28]	1 × 10^-16^

Values are mean with (relative standard deviation %) or [95% confidence interval].

The force needed to displace the catheter away from its needle in the 22G Cannula P was 362% higher than Cannula I (p < 0.001), 35% higher in the 20G (p = 0.12) and 445% higher in the 18G (p < 0.001). These results are summarised in Table 2. With respect to precision, there was significant variability with both Cannula I and P having relative standard deviations more than 10%.

**Table 2:** Measurements of force required to displace the catheter from the needle in Cannula P and I as an indirect measure of static resistance

	**Cannula P**	**Cannula I**	**Difference**	**p-value**
22G static resistance; N	1.80 (25.2)	0.39 (28.7)	1.41 [1.09 to 1.73]	2 × 10^-6^
20G static resistance; N	0.73 (40.0)	0.55 (37.3)	0.19 [-0.04 to 0.41]	0.12
18G static resistance; N	2.40 (37.4)	0.44 (13.3)	1.96 [1.40 to 2.52]	7 × 10^-5^

Values are mean with (relative standard deviation %) or [95% confidence interval].

The two-week trial period occurred in April 2013. Of the 19 electronic surveys sent, 17 were returned, a response rate of 89%. Of the 17 anaesthetists, 16 (94%) had trialled both Cannula P and I. 9 out of 16 responses (56%) labelled Cannula P “significantly worse” than Cannula I. 13 out of 17 responses (76%) preferred using Cannula I over P. Responses are summarised in Table 3. The fact that Cannula P had higher resistance through its needle compared to Cannula I was noted in the comments section through descriptions of “slow” or “delayed” flashback. This was seen as the primary cause of inadvertent puncture through the vein and the requirement for multiple attempts. Other comments were consistent with the finding that the force required to displace the catheter in Cannula P was higher compared to Cannula I.

**Table 3:** Survey responses from consultant anaesthetists regarding the use of Cannula P and I

**Question**	**n (%)**
Have you used both Cannula P and I? (n = 17)	
Yes	16 (94%)
No	1 (6%)
How does Cannula P compare to Cannula I? (n = 16)	
Significantly better	0 (0%)
Better	0 (0%)
No difference	0 (0%)
Worse	7 (44%)
Significantly worse	9 (56%)
What should we do? (n = 17)	
Use Cannula P over I	0 (0%)
Use either Cannula P or I	1 (6%)
Use Cannula I over P	13 (76%)
Stop using both Cannula P and I	1 (6%)
Stop using all safety cannulae	2 (12%)

The main difference of Cannula P’s needle was that it is was on average 17% longer than Cannula I’s. The length was 6.3 cm in Cannula P versus 5.4 cm in Cannula I in the 22G, and 7.2 cm versus 6.15 cm in both the 20G and 18G. The casing surrounding the needles in Cannula P are also frosted compared to Cannula I which are clear. This was noted as making it “difficult to visualise flashback” in the comments section. Other comments regarding design included the absence of a tab on Cannula P’s catheter compared with Cannula I, making it difficult to advance. The “lack of grip” on Cannula P’s catheter was also thought to contribute to this. There were minimal differences in the reported physical characteristics of catheters by the manufacturers (Table 4).

**Table 4:** Manufacturer specified physical characteristics of catheters

		**Cannula P**	**Cannula I**
18G (1.3 mm)	Length (mm)	32	32
	Flow rate (ml.min^-1^)	95	105
20G (1.1 mm)	Length (mm)	32	32
	Flow rate (ml.min^-1^)	65	60
22G (0.9 mm)	Length (mm)	25	25
	Flow rate (ml.min^-1^)	36	35

## Discussion

To help guide departmental purchasing of intravenous cannulae we compared two proposed brands of cannula with an in-vitro performance study and a staff survey. We found that one cannula brand had greater flow in the needle and required less force to pull the cannula off the needle. This cannula brand was universally preferred in the staff survey. With experience, this selection process has the potential to reduce waste and unnecessary human trials if the product is deemed unsuitable during the laboratory testing phase. This has ethical considerations as cannulation is often a painful procedure associated with anxiety, distress and discomfort for the patient [4]. Our study demonstrates that better regulation is warranted for medical devices, similar to the requirements for medicines [8].

We agree with Wilkes et al. that a centralised structured and coordinated approach to assess devices is needed [7]. This includes the establishment of a device evaluation centre run by a panel of experts that critically appraises the evidence, design laboratory based assessments, co-ordinates clinical studies, and makes recommendations on which devices are suitable for use [7]. We believe this could easily be operated and funded from a professional body that represents anaesthetists such as the The Royal College of Anaesthetists. A purchasing group within each hospital can then be established to trial a selection of products considered to be non-inferior within defined limits, and choose one that reflects local needs. By removing inferior products from the market early, this structure will help guide manufacturers in appropriately designing their products to meet technical standards that have not been previously defined by ISO.

The design aims of the needle should be to minimise: 1) slow flashback; 2) sticking of catheter to the needle; 3) difficulty in threading the catheter; 4) blood spillage; and 5) pain on insertion. Points 1 to 3 are associated with difficult IV cannulation [5,6]. Currently, the ideal flow rate or time to flashback is unknown. We know that time to flashback increases as the IVC gauge increases (diameter decreases) if the length of the needle remains the same. This explains why higher gauge cannulae (smaller diameter) use shorter needle lengths to compensate. However faster flashback does not always correlate with higher success rates. A laboratory study by Treuren et al. [9] on 22G cannulae showed that despite one brand having the fastest flashback, it ranked second out of three in success. It is interesting to speculate why this may be so. We believe the main reason is that a rapid flashback leads to an underestimation that the whole bevel (tip to heel) has penetrated the vein. This suggests that when designing needle lengths and diameters, a balance needs to be struck between flashback that is too rapid or too slow.

Another factor in difficult cannulation is the sticking of the catheter to the needle [5,6]. This occurs more frequently with safety compared to non-safety cannulae [10], and active compared to passive safety cannulae [6,11]. However, another study showed no difference between active safety and non-safety cannulae [12]. A possible reason for this conflicting evidence is the variation in the static friction between the catheter and needle even within the same brand as shown in Table 2. This reflects a lack of precision in the current manufacturing process for both Cannula P and I. The presence of a tab or small wings on the catheter itself can also assist with its handling. Neither are present in the design of Cannula P, and from our survey responses, it is another design feature which should be included in all IVC.

Variability of physical characteristics within the same class of IVC can be clinically important because of its implications to patient welfare. While ISO does not have any recommendations for flow rates through the needle or static resistance between the catheter and needle, it does have recommendations of the acceptable ranges of flow rate through a catheter. For catheters with an outside diameter less than 1.0 mm (22G and above) the flow rate must be between 80 to 125% of that stated by the manufacturer [3]. For catheters with an outside diameter 1.0 mm or greater (20G and below), the flow rate must be between 90 to 115% of that stated by the manufacturer [3]. More research needs to be done to link the ideal physical characteristics to optimal performance during cannulation.

There are several limitations to our study. Firstly, it is impossible to blind anaesthetists to the different designs of the cannulae. This design bias means that innovation resistance may have been a factor. This is a concept in economic psychology [13] and is not well explored in medicine. The components to innovation resistance are: 1) degree of change required and 2) conflicts with the health professional’s current belief structure [14]. We addressed these considerations by designing objective blinded randomised measurements to correlate with our subjective survey. This showed that the quantitative component (higher resistance and higher static friction) matched the qualitative component of our survey (slow flashback and sticking of the catheter to the needle) which resulted in a clear preference of one cannula over another. Secondly, we did not compare rates of thrombophlebitis in the perioperative period. The catheter material (polyurethrane) in both Cannula I and P were however the same, with polyurethrane having a much lower rate of thrombophlebitis than teflon [15]. Finally, our survey did not quantify failure rates. It also did not categorise the opinions on the head to head use between the different gauges of Cannula I and P. There is therefore a possibility that the overall negative opinions of Cannula P could have been affected by just one of its gauges – with the most likely culprit being the 22G which was found to have the highest resistance and the second highest static friction. Further trials are needed to validate laboratory results with individual failure rates of the different gauges, which should also take into account the experience and skill of the anaesthetist.

## Conclusion

In conclusion, our study demonstrates that there are currently wide variations in the physical designs and attributes between different brands IVC despite meeting ISO standards. To assist purchasing, benchmarks such as those reported for catheters, should be tested for the needle: i) length; ii) radius; iii) time to flashback at a given pressure. Hospitals thinking of trialling different IV cannulae can investigate important physical characteristics that impact on cannulation success rates using the simple setup described in this paper and combine these results with a survey to guide purchasing decisions. The introduction of these procedures into clinical practice can potentially assist efficient product selection, reduce waste and avoid unnecessary human trials.

## Competing interests

The authors declare that they have no competing interests.

## Authors’ contributions

ST and BS designed the trial. KM and JG obtained the data. All six authors were involved in the analysis of the data and preparation of the manuscript. All authors read and approved the final manuscript.

## Pre-publication history

The pre-publication history for this paper can be accessed here:

http://www.biomedcentral.com/1471-2253/13/49/prepub
